# Repair of DNA Strand Breaks in a Minichromosome In Vivo: Kinetics, Modeling, and Effects of Inhibitors

**DOI:** 10.1371/journal.pone.0052966

**Published:** 2013-01-30

**Authors:** Slawomir Kumala, Krzysztof Fujarewicz, Dheekollu Jayaraju, Joanna Rzeszowska-Wolny, Ronald Hancock

**Affiliations:** 1 Laval University Cancer Research Centre, Hôtel-Dieu Hospital, Québec, Canada; 2 Bioinformatics Group, Institute of Automatic Control, Silesian University of Technology, Gliwice, Poland; 3 Biosystems Group, Institute of Automatic Control, Silesian University of Technology, Gliwice, Poland; Universita’ di Milano, Italy

## Abstract

To obtain an overall picture of the repair of DNA single and double strand breaks in a defined region of chromatin in vivo, we studied their repair in a ∼170 kb circular minichromosome whose length and topology are analogous to those of the closed loops in genomic chromatin. The rate of repair of single strand breaks in cells irradiated with γ photons was quantitated by determining the sensitivity of the minichromosome DNA to nuclease S1, and that of double strand breaks by assaying the reformation of supercoiled DNA using pulsed field electrophoresis. Reformation of supercoiled DNA, which requires that all single strand breaks have been repaired, was not slowed detectably by the inhibitors of poly(ADP-ribose) polymerase-1 NU1025 or 1,5-IQD. Repair of double strand breaks was slowed by 20–30% when homologous recombination was supressed by KU55933, caffeine, or siRNA-mediated depletion of Rad51 but was completely arrested by the inhibitors of nonhomologous end-joining wortmannin or NU7441, responses interpreted as reflecting competition between these repair pathways similar to that seen in genomic DNA. The reformation of supercoiled DNA was unaffected when topoisomerases I or II, whose participation in repair of strand breaks has been controversial, were inhibited by the catalytic inhibitors ICRF-193 or F11782. Modeling of the kinetics of repair provided rate constants and showed that repair of single strand breaks in minichromosome DNA proceeded independently of repair of double strand breaks. The simplicity of quantitating strand breaks in this minichromosome provides a usefull system for testing the efficiency of new inhibitors of their repair, and since the sequence and structural features of its DNA and its transcription pattern have been studied extensively it offers a good model for examining other aspects of DNA breakage and repair.

## Introduction

The molecular events implicated in repair of strand breaks in DNA are becoming more clear (reviewed in [1]–[6]), but an overall and quantitative picture of their repair in vivo which would contribute to understanding the systems biology of repair and the effects of inhibitors is not yet available. Current methods do not allow simultaneous and precise quantitation of repair of single and double strand breaks. Repair of double strand breaks, which are believed to be the crucial lesions leading to cell death [7], is commonly assayed by restoration of the normal length of genomic DNA or restriction fragments using pulsed-field gel electrophoresis (PFGE) [8]–[10]. Repair of single strand breaks, which may contribute to loss of viability by relaxing superhelical stress in genomic DNA loops and thus arresting transcription [11], cannot yet be quantitated specifically by methods with comparable precision.

As a model system to approach this question we are studying the repair of strand breaks in vivo in a ∼170 kb circular minichromosome, the Epstein-Barr virus (EBV) episome, which is maintained in the nuclei of Raji cells at 50–100 copies localised at the periphery of interphase chromosomes [12]–[17]. Two features of this minichromosome make it an attractive model for genomic chromatin: it can be considered as a defined region of chromatin in view of its canonical nucleosomal conformation [13] and the well-studied sequence and properties of its DNA [14], and its closed circular topology and length resemble those of the constrained loops which genomic chromatin forms in vivo [11], [18], [19]. After irradiating cells with ^60^Co γ photons we assayed the repair of single strand breaks in the minichromosome by quantitating the loss of nuclease S1-sensitive sites, and the repair of double strand breaks by PFGE assays of the reformation of supercoiled DNA from molecules which had been linearised. Circular molecules containing single strand breaks could not be quantitated directly, and instead their levels were calculated using a mathematical model developed to fit the experimental data. We exploited the possibility of quantitating repair in this system to examine the implication of particular enzymes, particularly topoisomerases I and II whose participation in repair has long been controversial [20]–[24], poly(ADP-ribose) polymerase-1 (PARP-1) [25]–[32], Rad51 [33], the catalytic subunit of DNA-protein kinase (DNA-PKcs) [2]–[6], [34], and ATM kinase [2]–[6], [35], [36]. New features of the repair of strand breaks in vivo and of their kinetics were revealed by mathematical modeling.

## Results

### Strand Breaks in the Minichromosome in Irradiated Cells

**Figure 1:**
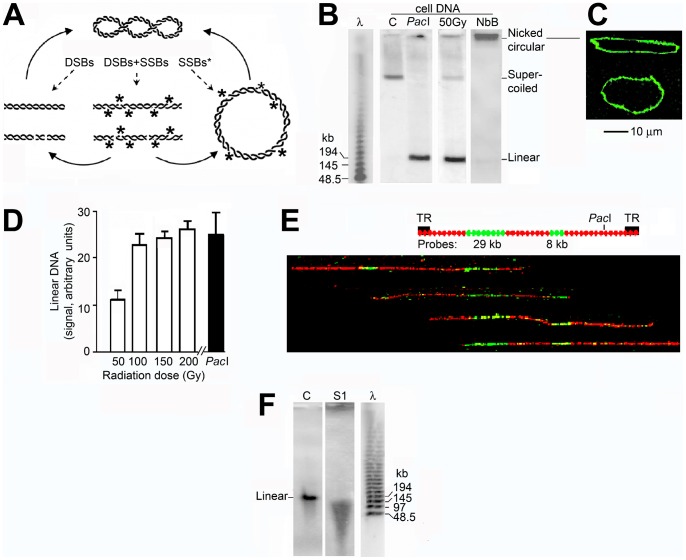
Strand breaks in minichromosome DNA in irradiated cells. (A) Supercoiled minichromosome DNA and forms which result from strand breaks. (B) Minichromosome DNA separated by PFGE after incubating deproteinised cells with: lane C, no addition; lane PacI, PacI (100 u/ml, 3 h) which cuts minichromosome DNA at a single site; lane NbB, endonuclease Nb.BbvCI (100 u/ml, 1 h) which forms circular molecules containing single strand breaks. Lane 50 Gy, cells irradiated (50 Gy) before deproteinisation; lane λ, oligomers of λ DNA. The gel was hybridised with a probe of EBV DNA; for the gel images in this and following Figures the top includes the sample well and panels were assembled from lanes of the same gel. (C) Representative DNA molecules believed to be relaxed circular minichromosome DNA containing single-strand breaks, extracted from the region close to the origin of a gel of DNA from cells incubated with endonuclease Nb.BbvCI (panel B, lane NbB), stained with YOYO-1, and combed (see text). (D) Quantitation of linear minichromosome DNA in irradiated cells compared with that after cleavage at its single PacI site (100 u/ml, 3 h) in deproteinised cells; error bars show SEM from three independent experiments. (E) Representative linear minichromosome DNA from irradiated cells spread by molecular combing and hybridised with the two probes shown on the upper map; TR are the terminal repeat sequences by which the minichromosome is circularised. The probes were labeled with biotin and detected with anti-biotin antibodies (green), and DNA was labelled with BrdU and detected with anti-BrdU antibodies (red). The extremities of the molecules show the site of the double strand break; the probe positions were aligned approximately considering the slightly variable stretching of DNA during combing. (F) Linear minichromosome DNA from irradiated cells extracted from a gel, incubated without or with nuclease S1 (100 u/ml, 15 h), and subjected to PFGE.

The supercoiled minichromosome DNA [12] and the forms which were expected to be produced in irradiated cells (linear, linear fragments, and nicked circular; Figure 1A) were quantitated by hybridising PFGE gels of total cell DNA with a probe of EBV DNA, the linear form of the minichromosome DNA [14] (Figure 1B). Nicked circular minichromosome DNA formed by incubating deproteinised cells with the nicking endonuclease Nb.BbvCI migrated diffusely between the sample well and the supercoiled form (Figure 1B), probably as a result of impalement on agarose fibres like other large nicked-circular DNAs [37]–[39]. Molecular combing of DNA from this region showed circular molecules 181±11 kb in length (SEM from 30 molecules) with the conformation expected for nicked circles (Figure 1C); these were not seen in DNA from untreated cells and did not have the theta conformation characteristic of replicating minichromosome DNA [40], while supercoiled DNA does not bind to slides in these conditions ([41] and data not shown). Because this region was diffuse and poorly separated from the sample well and may also contain replicating DNA molecules [37], we did not attempt to quantitate nicked circular molecules directly and instead calculated their abundance by mathematical modeling.

In irradiated cells the minichromosome DNA was converted to a form whose length, measured by interpolation from markers, was 170±10 kb (SEM from three independent experiments), a value not significantly different from that of full-length linear DNA (∼172 kb) (Figure 1B, lane 50 Gy). The amount of this DNA was not significantly different from that when minichromosome DNA was cut at its single PacI site (p = 0.45 from three replicate experiments) (Figure 1D). FISH on combed linear DNA molecules [42] from irradiated cells showed that their extremities were in variable positions with respect to two specific probes (Figure 1E). Together, these results show that the minichromosome DNA was converted quantitatively to full-length linear DNA in irradiated cells by one double strand break whose position was not specific [43]. Minichromosome DNA molecules which had been linearised by a double strand break were cleaved to shorter fragments by the single strand-specific nuclease S1 [44], [45] and therefore contained multiple single strand breaks (Figure 1F). The mean length of the S1 nuclease fragments did not decrease further when the concentration of nuclease was increased (data not shown).

### Repair of Strand Breaks

**Figure 2:**
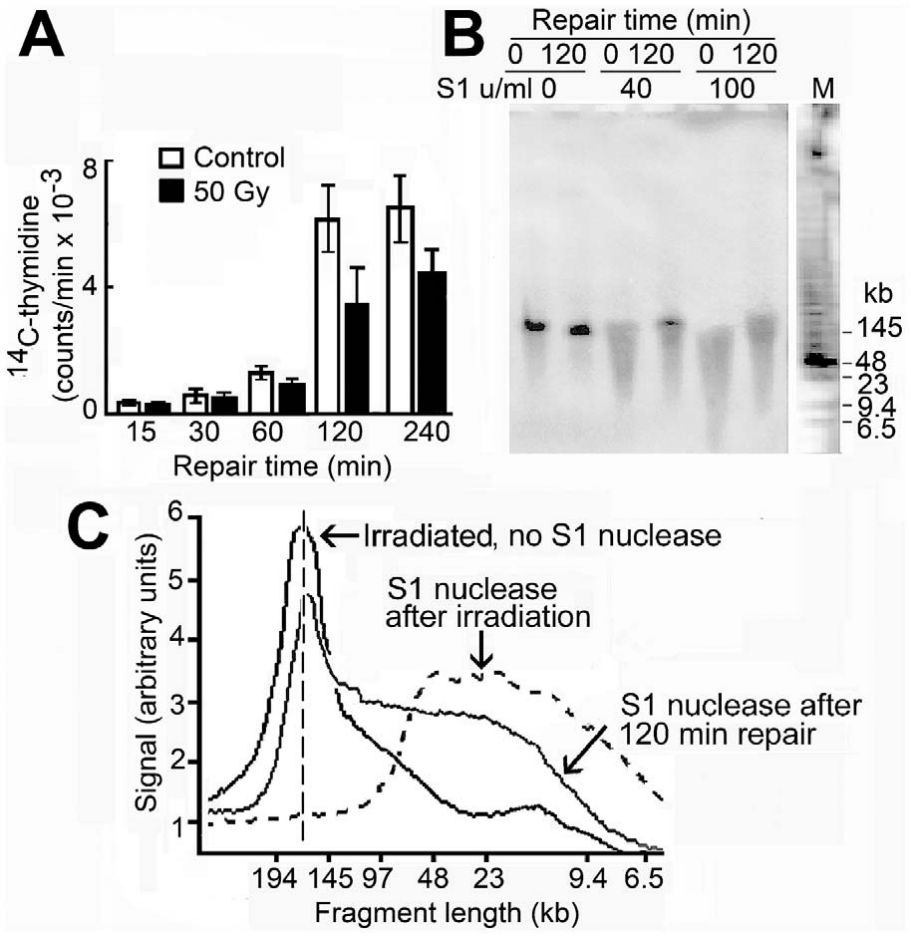
Repair of single strand breaks in linear minichromosome DNA. (A) DNA synthesis (incorporation of [^14^C]thymidine) in irradiated and control cells in the conditions used for repair; error bars show SEM from three independent experiments. (B) Fragmentation by nuclease S1 of linear minichromosome DNA isolated immediately after irradiation (50 Gy) or after repair for 2 h. Linear DNA was isolated from a gel of total cell DNA and incubated without or with nuclease S1 for 15 h and the fragments produced were separated by PFGE. For these experiments sufficient linear DNA could be conserved for 2 h only if repair of double strand breaks was arrested; this was achieved by including the DNA-PK inhibitor NU7441 during repair as described in the Section "Pathways for repair of double strand breaks". (C) Scans of the hybridisation signal from lanes in (B) (nuclease S1 100 u/ml); the position of full-length linear molecules is indicated by the vertical dashed line.

To quantitate repair rates precisely, the maximum conversion of minichromosome DNA to the linear form was desirable and cells were irradiated with 50 Gy, a dose similar to those commonly used to study repair of genomic DNA (for example [46], [47]). In the conditions used for repair, irradiated cells continued to synthesise DNA (Figure 2A). The single strand breaks in linear minichromosome DNA were repaired progressively (Figure 2B, C). Immediately after irradiation essentially all these molecules were cut by S1 nuclease to fragments of average length ∼20 kb, consistent with an average of 8 to 9 single strand breaks in each ∼172 kb molecule, while after 2 h of repair ∼50% of the molecules had been converted to the full-length linear form resistant to this nuclease and therefore contained no single strand breaks (Figure 2C).

### Repair of Double Strand Breaks and Recircularisation of Minichromosome DNA

**Figure 3:**
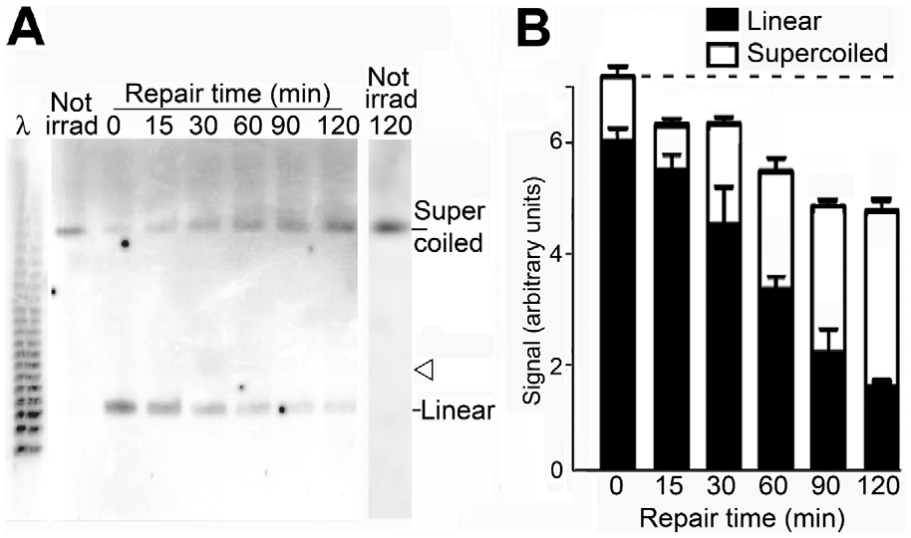
Repair of double strand breaks shown by the conversion of linear to supercoiled minichromosome DNA. (A) Linear and supercoiled DNA during repair; the arrowhead shows the calculated position of linear dimers which would have been formed by incorrect end-joining. (B) Linear (black columns) and supercoiled (white columns) minichromosome DNA quantitated by hybridisation; error bars show SEM from three independent experiments. The horizontal dashed line shows the level of linear plus supercoiled minichromosome DNA before repair.

During incubation for repair, supercoiled DNA accumulated progressively in parallel with a decrease of the linear form (Figure 3), showing that the double strand breaks by which linear molecules had been formed were religated. The sum of the linear and supercoiled forms decreased during incubation, consistent with an increase of the number of molecules which had been recircularised but still contained single strand breaks and were not quantitated directly. There was no evidence that minichromosome DNA was lost due to cleavage by endogenous or apoptotic nucleases during the repair period (see Discussion). Linear dimers of minichromosome DNA which would have been formed by incorrect end-joining were not detected (Figure 3A).

### Effect on Repair of Inhibiting Topoisomerases I and II

**Figure 4:**
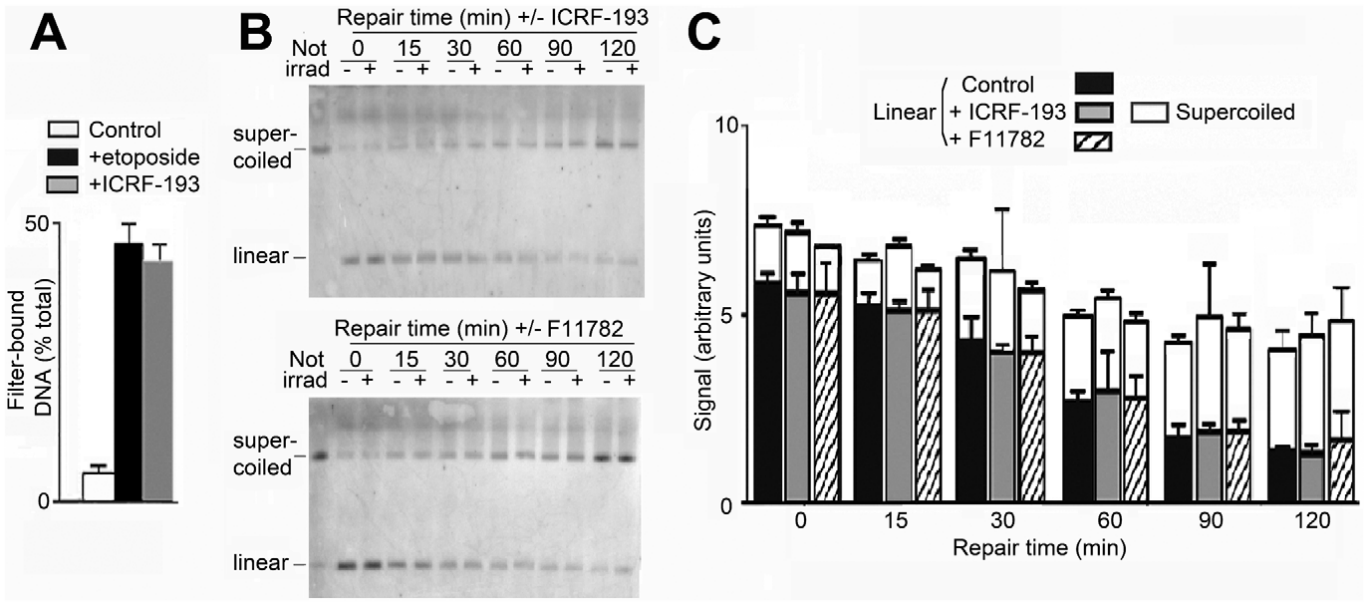
Conversion of linear to supercoiled DNA is not affected when topoisomerase II or both topoisomerases I and II are inhibited. (A) Efficiency of ICRF-193 (100 µM) in inhibiting topoisomerase II compared with that of the noncatalytic inhibitor etoposide (100 µM), assayed by quantitating covalent enzyme-DNA reaction intermediates in lysates of [^3^H]thymidine-labeled cells 1 h before incubation for repair. (B) Effect of ICRF-193 (100 µM) or F11782 (1 mM) on the conversion of linear to supercoiled DNA during repair. (C) Quantitation of linear and supercoiled DNA during repair. All error bars show SEM from three independent experiments.

The question if topoisomerases I and/or II are implicated in the repair of DNA strand breaks remains unresolved [20]–[24], [48]. We approached this question by inhibiting topoisomerases with inhibitors of the catalytic type which trap a noncovalent reaction intermediate and do not lead to cleavage of DNA after deproteinisation. To inhibit topoisomerase II we employed ICRF-193 [49]–[52] (100 µM), which was as efficient as etoposide in trapping reaction intermediates in cells [53] (Figure 4A); etoposide traps all cellular topoisomerase II at the concentration employed here [54]. The epipodophylloid F11782 [55]–[57] was used to inhibit both topoisomerases I and II; its efficiency in trapping enzyme-DNA reaction intermediates cannot be assayed [55] and we used a concentration of 1 mM which is >50-fold and >500-fold the IC_50_ for inhibition of human topoisomerases I and II, respectively, and >500-fold the IC_50_ for inhibition of growth of V79 cells [55].

Neither ICRF-193 nor F11782 had a significant effect on the evolution of the levels of linear and supercoiled minichromosome DNA during repair (Figure 4B, C). For supercoiled DNA the p-values for the difference in level after 2 h in the presence or absence of an inhibitor were 0.51 for ICRF-193 and 0.88 for F11782, and for linear DNA 0.71 and 0.51 respectively.

### Effect on Repair of Inhibiting PARP-1

**Figure 5:**
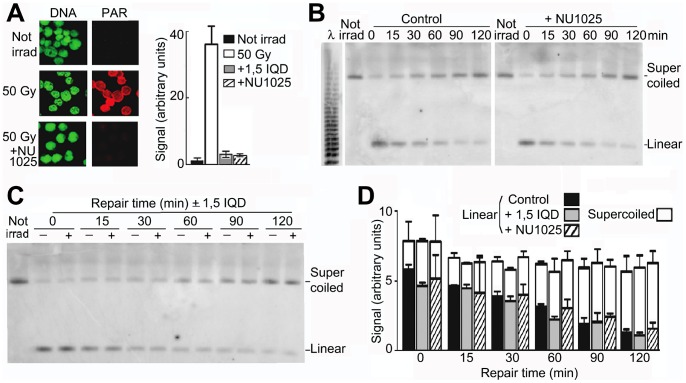
Conversion of linear to supercoiled DNA is not affected by inhibitors of PARP-1. (A) Activity of PARP-1 in cells incubated without or with NU1025 (200 µM), assayed by synthesis of PAR (red) immediately after irradiation; DNA was labeled with YOYO-1 (green). Right panel: quantitation of PAR (red pixel intensity/nuclear area); error bars show SEM from 200 nuclei. (B) Conversion of linear to supercoiled DNA in cells incubated alone or with NU1025 (200 µM) or (C) with 1,5-IQD (200 µM). (D) Quantitation of linear and supercoiled DNA during repair; error bars show SEM from four independent experiments.

PARP-1 has long been implicated in the sensing and repair of single strand breaks, but the step in which it participates has not yet been identified [25]–[32]. We inhibited PARP-1 by NU1025 [58] or 1,5-IQD [59] at a concentration of 200 µM; their IC_50_ values are 0.4 µM [58], [59]. The characteristic immediate synthesis of poly(ADP-ribose) (PAR) in irradiated cells was reduced by >95% by these inhibitors (Figure 5A). No detectable inhibition of repair of single strand breaks occurred since reformation of supercoiled DNA, which can only occur when all single strand breaks have been repaired, was not slowed (Figure 5B-D); the p-value for the difference in the level of supercoiled molecules at 120 min in the absence or presence of an inhibitor was 0.71 for NU1025 and 0.58 for 1,5-IQD.

### Pathways for Repair of Double Strand Breaks

**Figure 6:**
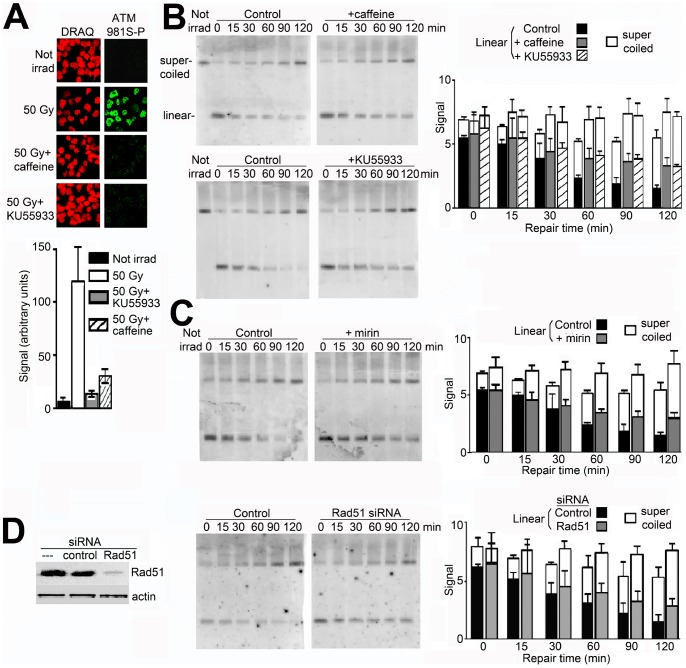
Effect of inhibiting HR-mediated repair of double strand breaks. (A) Phosphorylation of ATM on Ser1981 (green) in cells irradiated and incubated without or with caffeine (10 mM) or KU55933 (20 µM), assayed by immunofluorescence; DNA was stained by DRAQ (red). Below, quantitation of the signal from ATM1981S-P (green pixel intensity/nuclear area). (B) Repair of minichromosome DNA in cells incubated without or with caffeine (10 mM) or KU55933 (20 µM), inhibitors of ATM kinase, or (C) with mirin (100 µM) which prevents activation of ATM without affecting its kinase activity. (D) Repair in cells transfected with siRNA to silence expression of Rad51 or with a control siRNA; cells were irradiated 48 h later and incubated for repair. Rad51 protein was detected in cell lysates by Western blot, with actin as a sample loading control. All error bars show SEM from three independent experiments.

Double strand breaks in genomic DNA are repaired by two major pathways, homologous recombination (HR) and nonhomologous end-joining (NHEJ). The HR pathway is initiated by autophosphorylation of ATM on serine-1981 which initiates its kinase activity [2]–[6]. The inhibitor of ATM kinase KU55933 [60] reduced this phosphorylation in irradiated cells by ∼95%, while the inhibitor caffeine [61] reduced it by ∼80% (Figure 6A). In both cases, the rate of decrease of linear DNA showed a significant reduction of ∼30% (Figure 6B) (p<0.005 for KU55933, p<0.01 for caffeine). This rate was reduced by ∼26% in the presence of mirin (Figure 6C) which indirectly inhibits the activation of ATM without affecting its kinase activity [62], [63], and by ∼20% in cells where Rad51, which participates uniquely in HR [33], was depleted by ∼90% by a specific siRNA (Figure 6D). Together, these results are consistent in suggesting that 20–30% of the double strand breaks in the minichromosome were repaired by HR.

**Figure 7:**
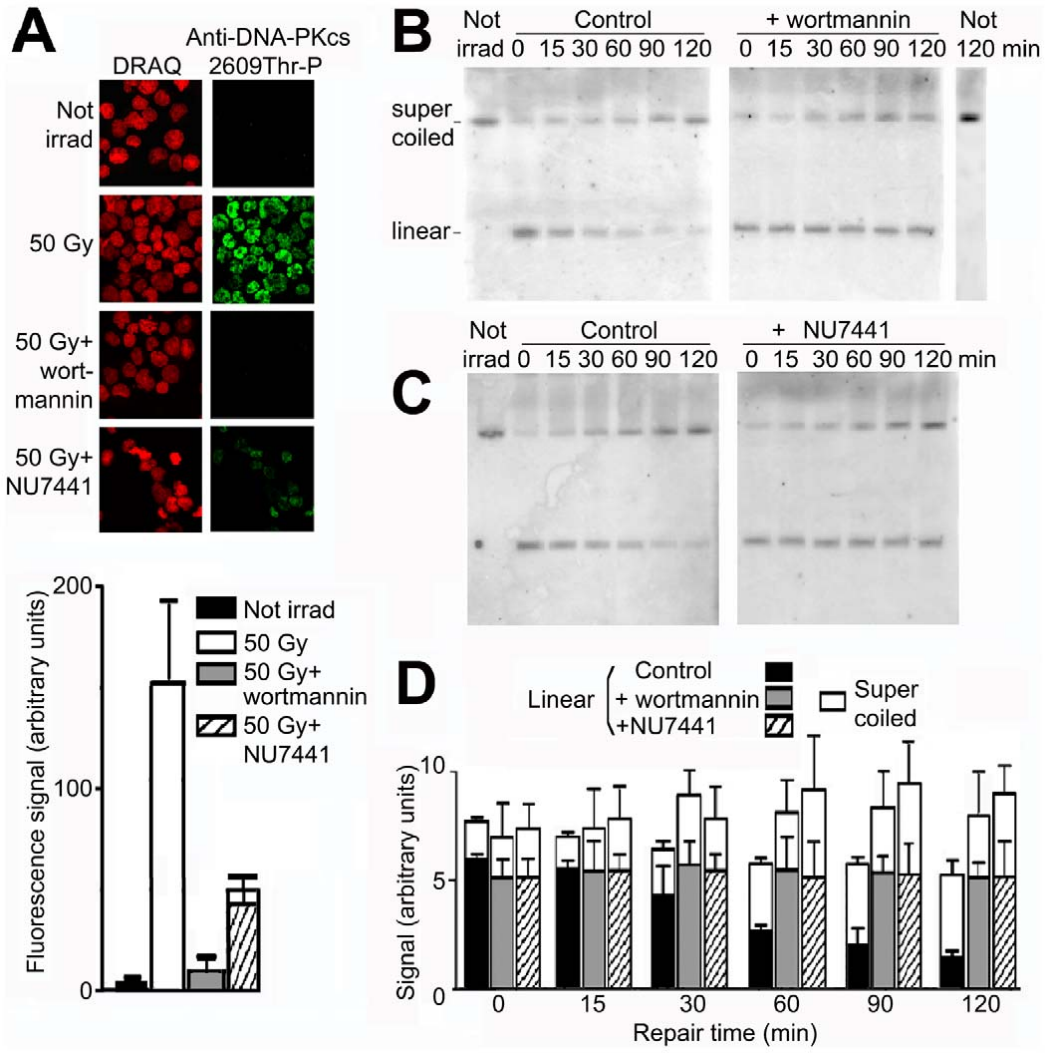
Arrest of double strand break repair by inhibitors of DNA-PKcs phosphorylation. (A) Phosphorylation of DNA-PKcs on threonine-2609 (green) in cells irradiated and incubated without or with wortmannin (100 µM) or (C) without or with NU7441 (10 µM) assayed by immunofluorescence; DNA was stained by DRAQ (red). Below, quantitation of the signal from DNA-PKcs2609Thr-P (green pixel intensity/nuclear area). (B) Repair in cells incubated with wortmannin (100 µM) or (C) NU7441 (10 µM). (D) Quantitation of linear and supercoiled DNA during repair. Error bars show SEM from three independent experiments, or two independent experiments for NU7441.

Repair of double strand breaks by the NHEJ pathway is initiated by binding of Ku70/Ku80 to DNA extremities, followed by recruitment of DNA-PKcs which is then activated by phosphorylation on threonine-2609 [2]–[6]. This phosphorylation was inhibited essentially completely by wortmannin [64] (p = 0.10 from two replicate experiments) and reduced by ∼70% by NU7441 [65] (Figure 7A). Both of these inhibitors completely arrested the repair of double strand breaks, as shown by the constant level of linear minichromosome DNA (Figure 7B–D) (p = 0.55 for wortmannin, p = 0.88 for NU7441). The formation of supercoiled DNA continued, reflecting ongoing repair of single strand breaks in circular molecules. The relative contributions of HR and NHEJ to the repair of double strand breaks are considered in the Discussion.

### Modeling the Kinetics of Strand Break Repair

**Figure 8:**
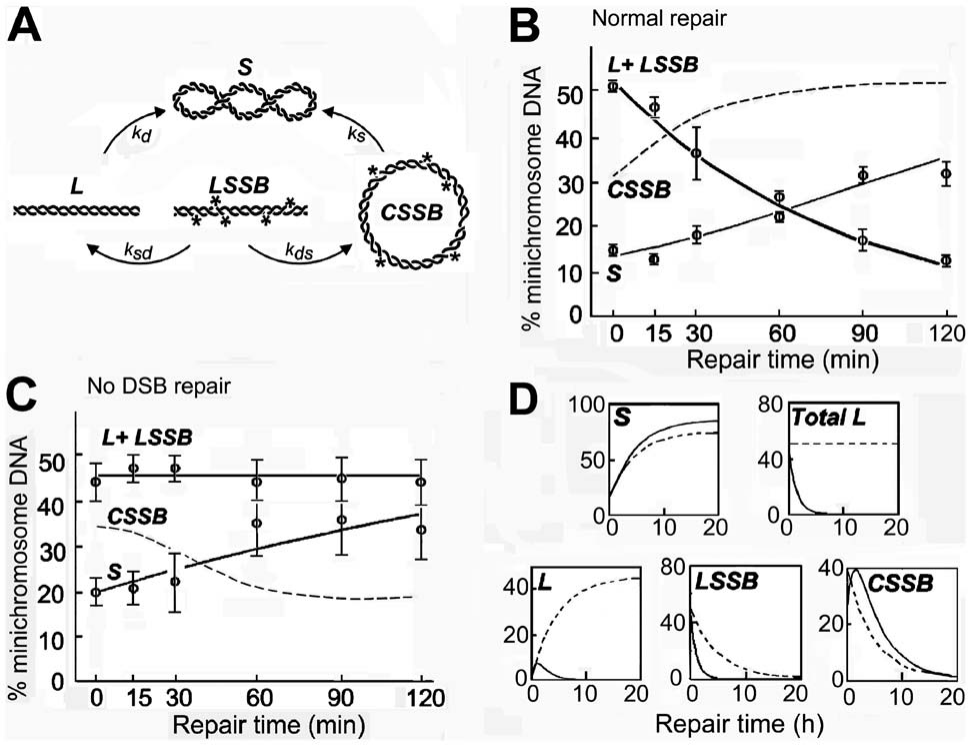
Temporal evolution of the levels of different forms of minichromosome DNA during repair calculated by modeling. (A) The model considered transfers of molecules between four compartments containing supercoiled molecules (*S*), linear molecules formed by a double strand break (*L*), linear molecules also containing single strand breaks (*) (*LSSB*), and circular molecules containing single strand breaks (*CSSB*). *k_s_, k_sd_, k_d_,* and *k_ds_* are the rate constants, and *k_d_,* and *k_ds_* were set at zero when repair of double strand breaks was arrested by the inhibitor NU7441. (B, C) Calculated levels of the different forms of minichromosome DNA (curves) together with the experimental data points with SEM from three independent experiments, (B) during normal repair or (C) when repair of double strand breaks is arrested. (D) Calculated levels of the different forms of DNA extrapolated for a period of 20 h in normal conditions (full lines) or when the repair of double strand breaks is arrested (dashed lines).

To compute the abundance of circular minichromosome DNA molecules which contained single strand breaks which could not be measured directly, a mathematical model was developed to fit the kinetics of repair (Figure 8A). This model offered the further advantage of providing rate constants for repair of strand breaks, as well as several conclusions which were not immediately evident from the experimental data (see Discussion). The interconversions of different forms of minichromosome DNA during repair were expressed by first-order kinetics; these require fewer parameters than Michaelis-Menten kinetics and if too many parameters are considered unique values cannot be calculated (the model is non-identifiable) and inferences are not reliable (see Discussion). Initially, the rate constants for repair of double strand breaks in molecules containing only a double strand break or also single strand breaks (*k_d_* and *k_ds_*) and for repair of single strand breaks in molecules with only these breaks or also a double strand break (*k_s_* and *k_sd_*) were assigned different values, but the fit to the data was not better than when identical values were used and the calculated parameters were too sensitive to the choice of starting point for optimisation. Identical values were therefore adopted for *k_d_* and *k_ds_* and for *k_s_* and *k_sd_*. The input data were the levels of linear and supercoiled DNA both during normal repair and when repair of double strand breaks was arrested by NU7441, when *k_d_* and *k_ds_* were set at zero. We underline that the calculated *k* values refer to the *fraction of the total molecules transferred between compartments per hour* and not to the *number of strand breaks repaired per hour*, and that they are therefore average values for molecules which contain single strand breaks because the number of these breaks varies in different molecules (Figure 2).

The calculated levels of the different forms of minichromosome DNA and their satisfactory fit to the experimental data are shown in Figure 8B and C. The estimated rate constant for complete repair of molecules which contained single strand breaks was *k_s_* = *k_sd_*  = 0.21 (95% confidence interval (CI) 0.16–0.27) and that for repair of molecules containing double strand breaks was *k_d_* = *k_ds_*  = 0.74 (95% CI 0.59–0.92).

If it was assumed that the rate constants during the first 2 h were maintained, the level of the different forms of minichromosome DNA could be predicted for a longer period of repair (Figure 8D). The relative quantity of linear DNA without single strand breaks was predicted to increase transiently while that of linear DNA with single strand breaks decreased, reflecting ongoing repair of these breaks. When repair of double strand breaks was inhibited, linear DNA without single strand breaks was predicted to accumulate as expected if the repair of single strand breaks continued. The level of the circular form containing single strand breaks was predicted to increase transiently as linear molecules containing single strand breaks were circularised before these breaks were repaired, and as expected this increase was not seen when repair of double strand breaks was inhibited.

## Discussion

The simultaneous repair of single and double strand breaks in a defined region of chromatin in vivo has not been studied previously using quantitative methods, to our knowledge. The methods used to detect strand breaks in earlier studies, filter elution or single-cell DNA electrophoresis, cannot provide absolute numbers of breaks and the reported rates were variable (for example [66], [67]). We used two conditions to ensure that strand breaks were quantitated accurately: for PFGE, DNA was deproteinised at room temperature because extra strand breaks are created at higher temperatures [68], and hybridisation was carried out in dried gels because the transfer of large DNA fragments onto membranes [9], [10] is not quantitative [69]. In another study [70] published while this manuscript was in preparation, a significant amount of minichromosome DNA remained in the sample well of PFGE gels and was interpreted as nicked circles, but here little or no DNA remained in the wells and nicked circular DNA migrated slowly into the gel, possibly reflecting methodological differences. A Poisson distribution of strand breaks was assumed in [70], but is not consistent with our finding that only one double strand break is formed in minichromosome DNA in irradiated cells (Figure 1 and [43]); this assumption is not supported strongly by experimental evidence and does not take into account the variable conformations and microenvironments of chromatin in the nucleus. Single or double strand breakage of minichromosome DNA by apoptotic or other endogenous nucleases did not appear to be significant during incubation of cells for repair. Supercoiled DNA in non-irradiated cells showed no significant decrease in its level between 0 h and 2 h (Figure 3A). In irradiated cells its level remained identical to that in control cells when topoisomerases or PARP were inhibited (Figures 4, 5), but its stability in the presence of putative repair inhibitors could not be measured since they influenced its reformation by repair pathways. The level of linear minichromosome DNA in irradiated cells remained constant when NHEJ was inhibited, with a p-value for the difference in level between 0 h and 2 h of 0.55 for wortmannin and 0.88 for NU7441 (Figure 7).

To inhibit enzymes involved in repair of strand breaks, we used chemical reagents whose specificity has been well established because in most cases siRNA methodology did not provide sufficient depletion of enzymes (for example, 50–60% depletion for Ku70 and DNA-PKcs; data not shown). In other studies depletion of PARP-1 [71], [72], DNA ligases [73], and topoisomerase II [74] was also less than complete and in some cases lethal [74]. Inhibitors of PARP-1 showed no effect on the repair of strand breaks in minichromosome DNA. The precise step in which PARP-1 intervenes in repair remains elusive; the current view is that it is not indispensable for repair of single strand breaks in genomic DNA [25]–[32], [75]–[77] and its role appears to be indirect, for example by binding to breaks and protecting them from further degradation [78]. In another study using our experimental system [70] published while this manuscript was in preparation, knockdown of PARP-1 did not significantly affect repair of single or double strand breaks.

A possible role for topoisomerases I or II in DNA repair has been examined in several studies [20]–[24], but in some cases noncatalytic topoisomerase inhibitors were employed which themselves create strand breaks when DNA is deproteinised [49] and therefore cannot provide evidence for a role of topoisomerases in repair. Topological considerations predict that if nucleosomes do not dissociate completely in the neighbourhood of a strand break, the negative superhelicity which results from DNA wrapping on their surface would be conserved in the nicked circular and linear forms. Thus after the repair of all breaks, the religated circular form would recover the negative superhelicity of the original circular minichromosome DNA. Our finding that the conversion of linear to supercoiled minichromosome DNA continues at the normal rate when topoisomerases I and II are inhibited by catalytic inhibitors is consistent with this scenario.

It appeared paradoxical at first view that repair of double strand breaks in the minichromosome was arrested completely by inhibition of NHEJ, while 20–30% of the breaks appeared to be repaired by HR as deduced from the effects of inhibiting activation or activity of ATM kinase or depleting Rad51. These findings can be interpreted plausibly by the mechanism which has been proposed to understand similar observations on repair of double strand breaks in genomic DNA, which is reported to be completely inhibited when NHEJ is arrested by the DNA-PKcs inhibitor wortmannin [79], [80]; trapping of factors involved in NHEJ at DNA extremities is suggested to prevent the access of factors required for HR [5], [81]–[83]. We underline, however, that the particular pathway of double strand break repair which is arrested when DNA-PKcs is inhibited does not influence the quantitative outcomes of our model of repair kinetics. In genomic DNA the fraction of double strand breaks repaired by HR varies in different cell types [84]–[87] and is predominant in lower eukaryotes, whose smaller genome may allow homologous chromosomes to find each other more easily than those in higher eukaryotes [88]. Similarly, HR may be favoured in the minichromosome due to the proximity of numerous replicating and daughter DNA molecules in replication compartments [16] whose limited volume would facilitate finding a region of sequence homology in a neighbouring molecule. Linear oligomers of minichromosome DNA were not detected during repair, as also observed during repair of a 3 Mb double-minute chromosome [46] and transfected plasmids [22], reflecting juxtaposition of the extremities of the broken DNA by Ku [2]–[6] and the RMX complex [89]; we propose that a further important factor is the crowded macromolecular environment in the nucleus [90] because crowding strongly favours DNA circularisation and ligation by ligases IIIb and IV-XRCC4 which participate in NHEJ [91].

Kinetic models of strand break repair can be constructed with different degrees of complexity, but theory shows that the least complex model is preferable to provide concrete predictions [92]. Our data were fitted well by using first-order kinetics (Figure 8A), and we consider that this strategy was justified since other datasets for DNA repair have been fitted satisfactorily by first-order kinetics (for example [8], [93]), which only deviate significantly from higher-order models after two half-times (that is, after repair of 75% of the strand breaks) [93]; further, theoretical arguments show that “multiple processes (which are not neccessarily first-order) may combine to produce kinetic behavior indistinguishable from first-order and. are more likely to exist when reactions occur in a complex environment” [94]. A number of conclusions which were not directly apparent from the experimental data illustrated the usefullness of modeling. First, when repair of double strand breaks was arrested, the single strand breaks in linear molecules were still repaired and circular molecules containing single strand breaks were converted to supercoiled molecules at close to the normal rate (Figure 8B) showing that the systems which repair single and double strand breaks operate independently, which has not been demonstrated previously as far as we are aware. Second, the calculated rate constants show that in an average linearised minichromosome the double strand break was repaired three to four times faster than all the single strand breaks, so that the rate-limiting step for complete repair of minichromosomes was the repair of single strand breaks. These repair rates cannot be compaired directly with those reported for genomic DNA where the methods used could not quantitate breaks directly, but comparisons can be made in terms of the half-time for repair which is independent of the radiation dose [95], [96] and of the length of the region considered [9]. In the minichromosome the calculated half-time for repair of the double strand break in each molecule was ∼40 min, which is within the range of 20 to 110 min reported for genomic DNA [9], [66], [95]. For repair of single strand breaks the half-time of ∼140 min for repairing 8 to 9 breaks per molecule (Figure 2C) was equivalent to an average of ∼16 min/break, which is within the range (10–30 min) reported for genomic DNA [66], [67], [97]–[99].

This minichromosome offers an simple experimental system for quantitative testing of potential inhibitors of repair of strand breaks, and since the sequence and structural features of its DNA and its transcription pattern have been studied extensively [14] it provides a good model for examining other facets of DNA breakage and repair, for example mapping strand breaks and comparing repair in transcribed and nontranscribed regions. Such studies may be relevant to the repair of DNA in genomic chromatin in view of the topological similarity of the minichromosome to chromatin loops and its position in regions of lower chromatin density within the nucleus [15], [17] where double strand breaks in genomic DNA and sites of their repair are predominantly localised [100], [101].

## Materials and Methods

### Cells, Irradiation, and Incubation for DNA Repair

Raji cells (an established cell line from L. Frappier, Department of Molecular Genetics, Toronto [102]) were grown in RPMI-1640 with 2 mM L-glutamine and 10% heat-inactivated FBS. Growing cells (0.5–1×10^6^) were washed in PBS, embedded in blocks of 1% low melting-point (LMP) agarose for PFGE, immersed in growth medium in closed 2 ml microtubes, and irradiated with ^60^Co γ photons (Teratron, Atomic Energy of Canada) at 4.3 Gy/min on ice. To follow DNA repair the blocks were transferred immediately into microplate wells containing growth medium at 37°C and placed in a CO_2_ incubator. DNA synthesis was followed by adding [methyl-^14^C]thymidine (1.5 kBq/ml) and taking samples into 5% TCA, collection on GF/B filters, washing with 5% TCA and 70% ethanol, and liquid scintillation counting. For incubation with restriction enzymes or endonuclease Nb.BbvCI (New England Biolabs) cells were encapsulated in beads of 1% LMP agarose [19], permeabilised in 10 mM Tris-HCl, 140 mM NaCl, 1 mM MgCl_2_, pH 7.6, 0.5% v/v Triton X-100 (Sigma-Aldrich), and washed 3×30 min in this buffer without Triton X-100.

### Inhibition of Enzymes Involved in Repair

Wortmannin and caffeine (Sigma-Aldrich), NU1025 and 1,5-IQD (Calbiochem), and NU7441, KU55933, and Mirin (Tocris) were dissolved in DMSO. ICRF-193 (gift of J. Nitiss, Molecular Pharmacology Department, St Jude Children’s Research Hospital, Memphis) and F11782 (gift of J-M. Barret, Centre de Recherche en Oncologie Expérimentale, Institut de Recherche Pierre Fabre, Toulouse) were dissolved in DMSO and H_2_O, repectively. Inhibitors were added to cultures 2 h before irradiation and to the medium after irradiation. Inhibition of topoisomerase II was assayed 1 h before incubation for repair in lysates of cells grown for 48 h with [methyl-^3^H]thymidine (37 kBq/ml) [103]. Inhibition of phosphorylation of DNA-PKcs or ATM was assayed using cells cytospun onto polylysine-coated slides, fixed in 4% formaldehyde in PBS for 15 min, permeabilised in PBS, 1% Triton X-100 (PBST) for 15 min, incubated in blocking solution (Boehringer) for 1 h and then with a mouse mAb recognising DNA-PKcs phosphorylated on threonine-2609 (Abcam, 1∶200) or ATM phosphorylated on serine-1981 (Cell Signaling, 1∶200) followed by Alexa 488-goat anti-mouse (1∶400). DNA was labeled with DRAQ5 (20 µM, 10 min) (Invitrogen). Poly(ADP-ribose) formation was detected using a rabbit polyclonal antibody (Alexis, 1∶50, overnight at 4°C) followed by Alexa 594-goat anti-rabbit IgG (1∶200, 30 min at 37°C); DNA was stained with YOYO-1 (1 µM, 10 min). Antibody dilutions and washings were in PBST and slides were mounted in SlowFade Gold (Invitrogen). Cells were imaged (Nikon E800, 40x objective) and total pixel intensities and areas were measured in 200 nuclei using MetaMorph 4.60 (Molecular Devices).

### Depletion of Rad51

Two×10^5^ cells in 50 µl serum- and antibiotic-free RPMI medium in wells of a 96-well dish were supplemented with 50 µl of a preincubated mixture containing 0.8 µl Oligofectamine (Invitrogen) and 100 pmol siRNA for Rad51 (siGenome SMART pool, Dharmacon) (this concentration of siRNA is required for efficient depletion of enzymes in Raji cells [104]) and incubated overnight at 37°C. Transfection efficiency was >85% as assayed using an FITC-labeled nonsilencing siRNA (Cell Signalling). Cells were irradiated after 48 h and incubated for repair. Rad51 protein was quantitated by lysing cells in SDS/PAGE sample buffer, SDS/PAGE, transfer to a nitrocellulose membrane, and probing with anti-Rad51 antibody (H-92, Santa Cruz) and anti-actin (C2) (Jackson ImmunoResearch) as loading control.

### PFGE, Probes, and Hybridisation

Agarose blocks were deproteinised in 1 ml 0.2 M EDTA, 1% SDS, 1 mg/ml proteinase K (Roche) for 48 h with rocking at ∼18°C; this procedure solubilised >99% of the 10% TCA-precipitable radioactivity from cells containing ^35^S-labelled proteins (data not shown). PFGE was in 1% agarose in 0.5X TBE at 14°C using 190 v for 20 h with pulse time increased linearly from 50 to 90 sec. Single strand breaks in linear minichromosome DNA were detected by excising the corresponding region from a gel, washing with S1 nuclease buffer, and incubation with S1 nuclease (Invitrogen) for 15 h at 37°C. Hybridisation was performed on gels placed on 3 MM paper, covered with plastic film, and dried under vacuum at 60°C for 1 h. Dried gels were incubated in 0.5 M NaOH, 1.5 M NaCl for 30 min, rinsed 3x in H_2_O, neutralised in 0.5 M Tris, pH 8.0, 1.5 M NaCl for 30 min, rinsed with H_2_O, and incubated in 6X SSC for 20 min, all at room temperature. Prehybridisation (30 min) and hybridisation (18 h) were in 6X SSC, 5X Denhardt’s solution, 0.5% SDS, 0.5 µg/ml human Cot-1 DNA (Invitrogen) at 68°C. The hybridisation probe was DNA of EBV virus (GenBank AJ507799) prepared from B95-8 cells (an established cell line from P. de Campos-Lima, Cancer Research Centre, Québec [105]) or a specific probe for marker lanes, labeled with [α-^32^P]dCTP (3000 MBq/mM) using Megaprime kits (Amersham). Hybridised gels were washed 3×30 min in 0.1X SSC, 0.5% SDS at 68°C, sealed in plastic film, and exposed to PhosphorImager screens. Signals were imaged, quantitated, and scanned using ImageQuant (Molecular Dynamics) and are shown as (10^−7^ x arbitrary intensity units) in the region of interest after subtracting the mean background in two identical adjacent areas. Samples from the same cell population without or with an inhibitor were processed in parallel, separated in the same gel, and when a central marker lane was excised the remaining parts of the gel were hybridised together. Repair rates were quantitated in replicate experiments and inhibition was expressed as the difference in level of forms of minichromosome DNA between cells with and without an inhibitor after 2 h. p-values were calculated by the unpaired t-test.

### Molecular Combing and Hybridisation of Minichromosome DNA

Linear minichromosome DNA from cells grown with BrdU was excised from PFGE gels in LMP agarose. The agarose was incubated with YOYO-1 (5 µM) for 30 min, washed in TE, incubated in β-agarase buffer for 30 min on ice, melted in 50 mM MES, pH 5.7 at 65°C for 10 min, and solubilised by β-agarase (New England Biolabs) at 42°C for 4 h. Four µl of DNA in the same buffer (∼2 µg/ml) were placed on a 3-aminopropyl-triethoxysilane-coated microscope slide (Sigma-Aldrich) and covered with a standard cover glass, which was pulled horizontally across the slide at ∼300 µm/sec after 2 min. Slides with well-spread DNA molecules as seen by fluorescence microscopy (Nikon E800, 100x objective) were dried at room temperature for 5 min, overnight at 60°C, incubated in 0.6X SSC, 70% formamide for 3 min at 95°C, and then in cold 70%, 85%, and 95% ethanol (2 min each). The probes were an 8.1 kb BamHI-SalI fragment of cosmid cM301-99 and a 29 kb HindIII fragment of cosmid cMB-14 (gifts from G. Bornkamm, Institute for Clinical Molecular Biology and Tumor Genetics, Munich) excised from an agarose gel, purified on a Microcon YM-100 (Qiagen), and labeled with biotin-11-dUTP (Fermentas) by nick translation. Hybridisation was in a humidified chamber at 37°C for up to 48 h. Probes were detected with FITC-goat anti-biotin (Sigma-Aldrich) (1∶50, 20 min) followed by Alexa 488-rabbit anti-goat antibody (Invitrogen) (1∶50, 20 min), and DNA by subsequent incubation with rat anti-BrdU (Abcam) (1∶30, 20 min) followed by Alexa 594-goat anti-rat antibody (Invitrogen, 1∶50, 20 min). Antibody dilutions and washing were in PBS, 0.05% Tween-20. Minichromosome DNA molecules identified by signals from both probes were imaged (Bio-Rad MRC1024 confocal) and their lengths were calculated using the factor of 2.2 kb DNA/µm after minor adjustment of images to normalise the distance between the two probes, as described in [42].

### Modeling Repair Kinetics

Four compartments each containing one form of minichromosome DNA were considered together with the four ordinary differential equations:
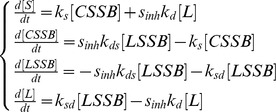
where:

[*X*] = fraction of total amount (hybridisation signal) of DNA in form *X.*

(S = supercoiled, L = linear, LSSB = linear with single strand breaks, CSSB  = circular with single strand breaks);

*k_d_* = rate of repair of molecules containing only a double strand break;

*k_s_* = rate of repair of molecules containing only single strand breaks;

*k_ds_* = rate of repair of the double strand break in molecules containing both a double and single strand breaks;

*k_sd_* = rate of repair of single strand breaks in molecules containing both single and a double strand break;

*s_inh_* = switch reflecting inhibition of double strand break repair: 1 for normal conditions, 0 when repair was arrested by the inhibitor NU7441 (Figure 7D).

The rationale for using first-order kinetics is considered in the Discussion. Fitting to the experimental data depended on estimating parameters and initial conditions in normal conditions or when double strand break repair was inhibited, using a least squares approach to minimise the sum of squared residuals (differences between data and the model’s output). Calculations were made in MATLAB.

## References

[1] Caldecott KW (2009) Chromosomal Single-Strand Break Repair. In: K.K. Khanna, Y. Shiloh, editors, The DNA Damage Response: Implications on Cancer Formation and Treatment. Springer, Berlin. 261–284.

[2] Hartlerode AJ, Scully R (2009) Mechanisms of double-strand break repair in somatic mammalian cells. Biochem. J 423: 157–168. 10.1042/BJ20090942 PMC2983087 19772495

[3] Lieber MR (2010) The mechanism of double-strand DNA break repair by the nonhomologous DNA end-joining pathway. Annu Rev Biochem 79: 181–211. 20192759 10.1146/annurev.biochem.052308.093131 PMC3079308

[4] Mahaney BL, Meek K, Lees-Miller SP (2009) Repair of ionising radiation-induced DNA double-strand breaks by non-homologous end-joining. Biochem J 417: 639–650. 19133841 10.1042/BJ20080413 PMC2975036

[5] Shrivastav M, Miller CA, De Haro LP, Durant ST, Chen BP, et al (2009) DNA-PKcs and ATM co-regulate DNA double-strand break repair. DNA Repair 8: 920–929. 19535303 10.1016/j.dnarep.2009.05.006 PMC3071034

[6] van Gent DC, van der Burg M (2007) Non-homologous end-joining, a sticky affair. Oncogene 26: 7731–7740. 18066085 10.1038/sj.onc.1210871

[7] Olive PL (1998) The role of DNA single- and double-strand breaks in cell killing by ionising radiation. Radiat Res 150: S42–51. 9806608

[8] Ahn SY, Nevaldine B, Hahn PJ (1991) Direct measurement by pulsed-field gel electrophoresis of induction and rejoining of X-ray-induced double-strand breaks in cultured mouse cells. Int J Radiat Biol 59: 661–675. 1672356 10.1080/09553009114550591

[9] Löbrich M, Rydberg B, Cooper P (1995) Repair of x-ray-induced DNA double-strand breaks in specific NotI restriction fragments in human fibroblasts: joining of correct and incorrect ends. Proc Natl Acad Sci USA 92: 12050–12054. 8618842 10.1073/pnas.92.26.12050 PMC40294

[10] Rydberg B, Löbrich M, Cooper PK (1994) DNA Double-Strand Breaks Induced by High-Energy Neon and Iron Ions in Human Fibroblasts. I. Pulsed-Field Gel Electrophoresis Method. Radiat Res 139: 133–141. 8052688

[11] Luchnik AN, Hisamutdinov TA, Georgiev GP (1988) Inhibition of transcription in eukaryotic cells by X-irradiation: relation to the loss of topological constraint in closed DNA loops. Nucleic Acids Res 16: 5175–5190. 3387223 10.1093/nar/16.11.5175 PMC336726

[12] Gussander E, Adams A (1984) Electron microscopic evidence for replication of circular Epstein-Barr virus genomes in latently infected Raji cells. J Virol 52: 549–556. 6092676 10.1128/jvi.52.2.549-556.1984 PMC254557

[13] Shaw JE, Levinger LF, Carter CW (1979) Nucleosomal structure of Epstein-Barr virus DNA in transformed cell lines. J Virol 29: 657–665. 219253 10.1128/jvi.29.2.657-665.1979 PMC353198

[14] Sugden B (2001) Leight (2001) ER EBV’s plasmid replicon, an enigma in cis and trans. Curr Top Microbiol Immunol 258: 3–11. 11443865 10.1007/978-3-642-56515-1_1

[15] Deutsch MJ, Ott E, Papior P, Schepers A (2010) The latent origin of replication of Epstein-Barr virus directs viral genomes to active regions of the nucleus. J Virol 84: 2533–2546. 20032186 10.1128/JVI.01909-09 PMC2820910

[16] Daikoku T, Kudoh A, Fujita M, Sugaya Y, Isomura H, et al (2004) In vivo dynamics of EBNA1-oriP interaction during latent and lytic replication of Epstein-Barr virus. J Biol Chem 279: 54817–54825. 15498777 10.1074/jbc.M405911200

[17] Kanda T, Kamiya M, Maruo S, Iwakiri D, Takada K (2007) Symmetrical localisation of extrachromosomally replicating viral genomes on sister chromatids. J Cell Sci 120: 1529–1539. 17405814 10.1242/jcs.03434

[18] Benyajati C, Worcel A (1976) Isolation, characterisation, and structure of the folded interphase genome of Drosophila melanogaster. Cell 9: 393–407. 825231 10.1016/0092-8674(76)90084-2

[19] Jackson DA, Dickinson P, Cook PR (1990) The sise of chromatin loops in HeLa cells. EMBO J 9: 567–571. 2303042 10.1002/j.1460-2075.1990.tb08144.x PMC551702

[20] Gaffney DK, Lundquist M, Warters RL, Rosley R (2000) Effects of modifying topoisomerase II levels on cellular recovery from radiation damage. Radiat Res 154: 461–466. 11023611 10.1667/0033-7587(2000)154[0461:eomtil]2.0.co;2

[21] Giocanti N, Hennequin C, Balosso J, Mahler M, Favaudon V (1993) DNA Repair and Cell Cycle Interactions in Radiation Sensitisation by the Topoisomerase II Poison Etoposide. Cancer Res 53: 2105–2111. 8386982

[22] Jacob S, Miquel C, Sarasin A, Pras F (2005) Effects of camptothecin on double-strand break repair by non-homologous end-joining in DNA mismatch repair-deficient human colorectal cancer cell lines. Nucleic Acids Res 33: 106–113. 15642697 10.1093/nar/gki154 PMC546142

[23] Mateos S, Hajji N, Pastor N, Cortés F (2006) Modulation of radiation response by inhibiting topoisomerase II catalytic activity. Mutat Res 599: 105–115. 16574164 10.1016/j.mrfmmm.2006.02.002

[24] Terry SY, Riches AC, Bryant PE (2009) Suppression of topoisomerase IIalpha expression and function in human cells decreases chromosomal radiosensitivity. Mutat Res 663: 40–45. 19428368 10.1016/j.mrfmmm.2009.01.003 PMC6175043

[25] Allinson SL, Dianova II, Dionov GL (2003) Poly(ADP-ribose) polymerase in base excision repair: always engaged, but not essential for DNA damage processing. Acta Biochim Pol 50: 169–179. 12673357

[26] Fisher AE, Hochegger H, Takeda S, Caldecott KW (2007) Poly(ADP-ribose) polymerase 1 accelerates single-strand break repair in concert with poly(ADP-ribose) glycohydrolase. Mol Cell Biol 27: 5597–5605. 17548475 10.1128/MCB.02248-06 PMC1952076

[27] Flohr C, Burkle A, Radicella JP, Epe B (2003) Poly(ADP-ribosyl)ation accelerates DNA repair in a pathway dependent on Cockayne syndrome B protein. Nucleic Acids Res 31: 5332–5337. 12954769 10.1093/nar/gkg715 PMC203308

[28] Godon C, Cordelières FP, Biard D, Giocanti N, Mégnin-Chanet F, et al (2008) PARP inhibition versus PARP-1 silencing: different outcomes in terms of single-strand break repair and radiation susceptibility. Nucleic Acids Res 36: 4454–4464. 18603595 10.1093/nar/gkn403 PMC2490739

[29] Noel G, Giocanti N, Fernet M, Megnin-Chanet F, Favaudon V (2003) Poly(ADP-ribose) polymerase (PARP-1–1) is not involved in DNA double-strand break recovery. BMC Cell Biol 4: 7. 12866953 10.1186/1471-2121-4-7 PMC179890

[30] Ström CE, Johansson F, Uhlén M, Al-Khalili Ssigyarto C, Erixon K, et al (2011) Poly(ADP-ribose)polymerase (PARP) is not involved in base excision repair but PARP inhibition traps a single-strand intermediate. Nucleic Acids Res 39: 3166–3175. 21183466 10.1093/nar/gkq1241 PMC3082910

[31] Veuger SJ, Curtin NJ, Smith GC, Durkacs BW (2004) Effects of novel inhibitors of poly(ADP-ribose) polymerase-1 and the DNA-dependent protein kinase on enzyme activities and DNA repair. Oncogene 23: 7322–7329. 15286704 10.1038/sj.onc.1207984

[32] Woodhouse BC, Dianova II, Parsons JL, Dianov GL (2008) Poly(ADP-ribose) polymerase-1 modulates DNA repair capacity and prevents formation of DNA double strand breaks. DNA Repair 7: 932–940. 18472309 10.1016/j.dnarep.2008.03.017

[33] Tambini CE, Spink KG, Ross CJ, Hill MA, Thacker J (2010) The importance of XRCC2 in RAD51-related DNA damage repair. DNA Repair 9: 517–525. 20189471 10.1016/j.dnarep.2010.01.016

[34] Neal JA, Dang V, Douglas P, Wold MS, Lees-Miller SP, et al (2011) Inhibition of homologous recombination by DNA-dependent protein kinase requires kinase activity, is titratable, and is modulated by autophosphorylation. Mol Cell Biol 31: 1719–1733. 21300785 10.1128/MCB.01298-10 PMC3126343

[35] Adams BR, Golding SE, Rao RR, Valerie K (2010) Dynamic Dependence on ATR and ATM for Double-Strand Break Repair in Human Embryonic Stem Cells and Neural Descendants. PLoS ONE 5: e10001. 20368801 10.1371/journal.pone.0010001 PMC2848855

[36] Beucher A, Birraux J, Tchouandong L, Barton O, Shibata A, et al (2009) ATM and Artemis promote homologous recombination of radiation-induced DNA double-strand breaks in G2. EMBO J 28: 3413–3427. 19779458 10.1038/emboj.2009.276 PMC2752027

[37] Beverley SM (1988) Characterisation of the ‘unusual’ mobility of large circular DNAs in pulsed field-gradient electrophoresis. Nucleic Acids Res 16: 925–939. 3344223 10.1093/nar/16.3.925 PMC334728

[38] Maleszka R (1993) Single-stranded regions in yeast mitochondrial DNA revealed by pulsed-field gel electrophoresis. Appl Theor Electrophor 3: 259–263. 8199217

[39] Wang M, Lai E (1995) Pulsed field separation of large supercoiled and open-circular DNAs and its application to bacterial artificial chromosome cloning. Electrophoresis 16: 1–7. 7737080 10.1002/elps.1150160102

[40] Adams A (1987) Replication of latent Epstein-Barr virus genomes in Raji cells. J Virol 61: 1743–1746. 3033303 10.1128/jvi.61.5.1743-1746.1987 PMC254169

[41] Allemand JF, Bensimon D, Jullien L, Bensimon A, Croquette V (1997) pH-dependent specific binding and combing of DNA. Biophys J 73: 2064–2070. 9336201 10.1016/S0006-3495(97)78236-5 PMC1181106

[42] Norio P, Schildkraut CL (2001) Visualization of DNA replication on individual Epstein-Barr virus episomes. Science 294: 2361–2364. 11743204 10.1126/science.1064603

[43] Kumala S, Hadj-Sahraoui Y, Rzeszowska-Wolny J, Hancock R (2012) DNA of a circular minichromosome linearized by restriction enzymes or other reagents is resistant to further cleavage: an influence of chromatin topology on the accessibility of DNA. Nucleic Acids Res Advance Access published July 30, 2012. doi:10.1093/nar/gks723. 10.1093/nar/gks723 PMC3479189 22848103

[44] Geigl EM, Eckardt-Schupp F (1990) Chromosome-specific identification and quantification of S1 nuclease-sensitive sites in yeast chromatin by pulsed-field gel electrophoresis. Mol Microbiol 4: 801–810. 2201869 10.1111/j.1365-2958.1990.tb00650.x

[45] Legault J, Tremblay A, Ramotar D, Mirault M-E (1997) Clusters of S1 nuclease-hypersensitive sites induced in vivo by DNA damage. Mol Cell Biol 17: 5437–5452. 9271420 10.1128/mcb.17.9.5437 PMC232393

[46] Nevaldine B, Riswana R, Hahn PJ (1999) No detectable misrejoining in double-minute chromosomes. Radiat Res 152: 154–159. 10409324

[47] Whitaker SJ, McMillan TJ (1992) Pulsed-Field Gel Electrophoresis in the Measurement of DNA Double-Strand Break Repair in xrs-6 and CHO Cell Lines:1594768

[48] Ng CE, Bussey AM, Raaphorst GP (1994) Inhibition of potentially lethal and sublethal damage repair by camptothecin and etoposide in human melanoma cell lines. Int J Radiat Biol 66: 49–57. 8027612 10.1080/09553009414550941

[49] D’Arpa P, Liu LF (1989) Topoisomerase-targeting antitumor drugs. Biochim Biophys Acta 989: 163–177. 2557085 10.1016/0304-419x(89)90041-3

[50] Ishida R, Miki T, Narita T, Yui R, Sato M, et al (1991) T Inhibition of intracellular topoisomerase II by antitumor bis(2,6-dioxopiperazine) derivatives: mode of cell growth inhibition distinct from that of cleavable complex-forming type inhibitors. Cancer Res 51: 4909–4916. 1654205

[51] Roca J, Ishida R, Berger JM, Andoh T, Wang JC (1994) Antitumor bisdioxopiperazines inhibit yeast DNA topoisomerase II by trapping the enzyme in the form of a closed protein clamp. Proc Natl Acad Sci USA 91: 1781–1785. 8127881 10.1073/pnas.91.5.1781 PMC43247

[52] Sato M, Ishida R, Narita T, Kato K, Ikeda H, et al (1997) Interaction of the DNA topoisomerase II catalytic inhibitor ICRF-193, a bisdioxopiperazine derivative, with the conserved region(s) of eukaryotic but not prokaryotic enzyme. Biochem Pharmacol 54: 545–550. 9337070 10.1016/s0006-2952(97)00201-3

[53] Gao H, Yamasaki EF, Chan KK, Shen LL, Snapka RM (2000) Chloroquinoxaline sulfonamide (NSC 339004) is a topoisomerase IIalpha/beta poison. Cancer Res 60: 5937–5940. 11085507

[54] Hsiang Y, Liu LF (1989) Evidence for the Reversibility of Cellular DNA Lesion Induced by Mammalian Topoisomerase II Poisons. J Biol Chem 264: 9713–9715. 2542330

[55] Etiévant C, Krucsynski A, Barret JM, Perrin D, van Hille B, et al (2000) F11782, a dual inhibitor of topoisomerases I and II with an original mechanism of action in vitro, and markedly superior in vivo antitumour activity, relative to three other dual topoisomerase inhibitors, intoplicin, aclarubicin and TAS-103. Cancer Chemother Pharmacol 4: 101–113. 10.1007/s002800000133 10972479

[56] Jensen LH, Renodon-Corniere A, Nitiss KC, Hill BT, Nitiss JL, et al (2003) A dual mechanism of action of the anticancer agent F 11782 on human topoisomerase II alpha. Biochem Pharmacol 66: 623–631. 12906927 10.1016/s0006-2952(03)00342-3

[57] Perrin D, van Hille B, Barret JM, Krucsynski A, Etiévant, et al (2000) F11782, a novel epipodophylloid non-intercalating dual catalytic inhibitor of topoisomerases I and II with an original mechanism of action. Biochem Pharmacol 59: 807–819. 10718339 10.1016/s0006-2952(99)00382-2

[58] Griffin RJ, Pemberton LC, Rhodes D, Bleasdale C, Bowman K, et al (1995) Novel potent inhibitors of the DNA repair enzyme poly(ADP-ribose) polymerase (PARP-1). Anti-Cancer Drug Design 10: 507–514. 7575991

[59] Banasik M, Komura H, Shimoyama M, Ueda K (1992) Specific Inhibitors of Poly(ADP-Ribose)synthetase and Mono(ADP-Ribosyl)transferase. J Biol Chem 267: 1569–1575. 1530940

[60] Hickson I, Shao Y, Richardson CJ, Green SJ, Martin NM, et al (2004) Identification and characterisation of a novel and specific inhibitor of the ataxia-telangiectasia mutated kinase ATM. Cancer Res 64: 9152–9159. 15604286 10.1158/0008-5472.CAN-04-2727

[61] Sarkaria JN, Busby EC, Tibbetts RS, Roos P, Taya Y, et al (1999) Inhibition of ATM and ATR kinase activities by the radiosensitising agent, caffeine. Cancer Res 59: 4375–4382. 10485486

[62] Williams RS, Williams JS, Tainer JA (2007) Mre11-Rad50-Nbs1 is a keystone complex connecting DNA repair machinery, double-strand break signaling, and the chromatin template. Biochem Cell Biol 85: 509–520. 17713585 10.1139/O07-069

[63] Dupré A, Boyer-Chatenet L, Sattler RM, Modi AP, Lee J, et al (2008) A forward chemical genetic screen reveals an inhibitor of the Mre11-Rad50-Nbs1 complex. Nature Chem Biol 4: 119–125. 18176557 10.1038/nchembio.63 PMC3065498

[64] Sarkaria JN, Tibbetts RS, Busby EC, Kennedy AP, Hill DE, et al (1998) Inhibition of phosphoinositide 3-kinase related kinases by the radiosensitising agent wortmannin. Cancer Res 58: 4375–4382. 9766667

[65] Leahy JJ, Golding BT, Griffin RJ, Hardcastle IR, Richardson C, et al (2004) Identification of a highly potent and selective DNA-dependent protein kinase (DNA-PK) inhibitor (NU7441) by screening of chromenone libraries. Bioorg Med Chem Lett 14: 6083–6087. 15546735 10.1016/j.bmcl.2004.09.060

[66] Mayer PJ, Bradley MO, Nichols WW (1986) No Change in DNA Damage or Repair of Single- and Double-strand Breaks as Human Diploid Fibroblasts Age In Vitro. Exp Cell Res 166: 497–509. 3743669 10.1016/0014-4827(86)90494-5

[67] Wlodek D, Hittelman WN (1987) The Repair of Double-Strand DNA Breaks Correlates with Radiosensitivity of L5178Y-S and L5178Y-R Cells. Radiat Res 112: 146–155. 3659295

[68] Stenerlöw B, Karlsson KH, Cooper B, Rydberg B (2003) Measurement of prompt DNA double-strand breaks in mammalian cells without including heat-labile sites: results for cells deficient in nonhomologous end joining. Radiat Res 159: 502–510. 12643795 10.1667/0033-7587(2003)159[0502:mopdds]2.0.co;2

[69] Leach TJ, Glaser RL (1998) Quantitative hybridisation to genomic DNA fractionated by pulsed-field gel electrophoresis. Nucleic Acids Res 26: 4787–4789. 9753752 10.1093/nar/26.20.4787 PMC147912

[70] Ma W, Halweg CJ, Menendez D, Resnick MA (2012) Differential effects of poly(ADP-ribose) polymerase inhibition on DNA break repair in human cells are revealed with Epstein-Barr virus. Proc Natl Acad Sci USA 109: 6590–6595. 22493268 10.1073/pnas.1118078109 PMC3340099

[71] Aneja RK, Sjodin H, Gefter JV, Singarelli B, Delude RL (2011) Small interfering RNA mediated Poly (ADP-ribose) Polymerase-1 inhibition upregulates the heat shock response in a murine fibroblast cell line. J Inflamm 8: 3. 10.1186/1476-9255-8-3 PMC3051880 21345219

[72] Audebert M, Salles B, Calsou P (2004) Involvement of poly(ADP-ribose) polymerase-1 and XRCC1/DNA ligase III in an alternative route for DNA double-strand breaks rejoining. J Biol Chem 279: 55117–55126. 15498778 10.1074/jbc.M404524200

[73] Windhofer F, Wu W, Iliakis G (2007) Low levels of DNA ligases III and IV sufficient for effective NHEJ. J Cell Physiol 213: 475–483. 17492771 10.1002/jcp.21120

[74] Johnson M, Phua HH, Bennett SC, Spence JM, Farr CJ (2009) Studying vertebrate topoisomerase 2 function using a conditional knockdown system in DT40 cells. Nucleic Acids Res 37: e98. 19494182 10.1093/nar/gkp480 PMC2724289

[75] Dantser F, de la Rubia G, Ménisser-De Murcia J, Hostomsky S, de Murcia G, et al (2000) Base excision repair is impaired in mammalian cells lacking Poly(ADP-ribose) polymerase-1. Biochemistry 39: 7559–7569. 10858306 10.1021/bi0003442

[76] Bowman KJ, White A, Golding BT, Griffin RJ, Curtin NJ (1998) Potentiation of anti-cancer agent cytotoxicity by the potent poly(ADP-ribose) polymerase inhibitors NU1025 and NU1064. Br J Cancer 78: 1269–1277. 9823965 10.1038/bjc.1998.670 PMC2063197

[77] Pachkowski BF, Tano K, Afonin V, Elder RH, Takeda S, et al (2009) Cells deficient in PARP1 show an accelerated accumulation of DNA single strand breaks, but not AP sites, over the PARP1-proficient cells exposed to MMS. Mutat Res 671: 93–99. 19778542 10.1016/j.mrfmmm.2009.09.006 PMC2784157

[78] Woodhouse BC, Dianov GL (2008) Poly ADP-ribose polymerase-1: An international molecule of mystery. DNA Repair 7: 1077–1086. 18468963 10.1016/j.dnarep.2008.03.009

[79] Boulton S, Kyle S, Yalçintepe L, Durkacz BW (1996) Wortmannin is a potent inhibitor of DNA double strand break but not single strand break repair in Chinese hamster ovary cells. Carcinogenesis 17: 2285–2290. 8968039 10.1093/carcin/17.11.2285

[80] Okayasu R, Suetomi K, Ullrich RL (1998) Wortmannin Inhibits Repair of DNA Double-Strand Breaks in Irradiated Normal Human Cells. Radiat Res 149: 440–445. 9588354

[81] Kim JS, Krasieva TB, Kurumisaka H, Chen DJ, Taylor AM, et al (2005) Independent and sequential recruitment of NHEJ and HR factors to DNA damage sites in mammalian cells, J Cell Biol. 170: 341–347. 10.1083/jcb.200411083 PMC2171485 16061690

[82] Sonoda E, Hochegger H, Saberi A, Taniguchi Y, Takeda S (2006) Differential usage of non-homologous end-joining and homologous recombination in double strand break repair. DNA Repair 5: 1021–1029. 16807135 10.1016/j.dnarep.2006.05.022

[83] Symington LS, Gautier J (2011) Double-strand break end reSection and repair pathway choice. Annu Rev Genet 45: 247–271. 21910633 10.1146/annurev-genet-110410-132435

[84] Shahar OD, Ram EV, Shimshoni E, Hareli S, Meshorer E, et al.. (2011) Live imaging of induced and controlled DNA double-strand break formation reveals extremely low repair by homologous recombination in human cells. Oncogene Nov 21. doi: 10.1038/onc.2011.516. [Epub ahead of print] 10.1038/onc.2011.516 22105360

[85] Orii KE, Lee Y, Kondo N, McKinnon PJ (2006) Selective utilisation of nonhomologous end-joining and homologous recombination DNA repair pathways during nervous system development. Proc Natl Acad Sci USA 103: 10017–10022. 16777961 10.1073/pnas.0602436103 PMC1502498

[86] Serrano L, Liang L, Chang Y, Deng L, Maulion C, et al (2011) Homologous Recombination Conserves DNA Sequence Integrity Throughout the Cell Cycle in Embryonic Stem Cells. Stem Cells Dev 20: 363–374. 20491544 10.1089/scd.2010.0159 PMC3128761

[87] Fung H, Weinstock DM (2011) Repair at Single Targeted DNA Double-Strand Breaks in Pluripotent and Differentiated Human Cells. PLoS ONE 6: e20514. 21633706 10.1371/journal.pone.0020514 PMC3102116

[88] Takata M, Sasaki MS, Tachiiri S, Fukushima T, Sonoda E, et al (2001) Chromosome instability and defective recombinational repair in knockout mutants of the five Rad51 paralogs. Mol Cell Biol 21: 2858–2866. 11283264 10.1128/MCB.21.8.2858-2866.2001 PMC86915

[89] Lobachev K, Vitriol E, Stemple J, Resnick MA, Bloom K (2004) Chromosome fragmentation after induction of a double-strand break is an active process prevented by the RMX repair complex. Curr Biol 14: 2107–2112. 15589152 10.1016/j.cub.2004.11.051

[90] Hancock R (2012) The crowded environment of the genome. In: K. Rippe, editor, Genome organisation and function in the cell nucleus. Wiley-VCH, Weinheim, 169–184.

[91] Chen L, Trujillo K, Sung P, Tomkinson AE (2000) Interactions of the DNA ligase IV-XRCC4 complex with DNA ends and the DNA-dependent protein kinase. J Biol Chem 275: 26196–26205. 10854421 10.1074/jbc.M000491200

[92] Nelson SJ (1982) Models for DNA Damage Formation and Repair in Mammalian Cells Exposed to Ionising Radiation. Radiat Res 92: 120–145. 7134379

[93] Fowler JE (1999) Is Repair of DNA Strand Break Damage from Ionising Radiation Second-Order Rather Than First-Order? A Simpler Explanation of Apparently Multiexponential Repair. Radiat Res 152: 124–136. 10409321

[94] Bandstra JS, Tratnyek PG (2005) Central limit theorem for chemical kinetics in complex systems. J Math Chem 37: 409–422.

[95] Foray N, Fertil B, Alsbeih MG, Badie C, Chavaudra N, et al (1996) Dose-rate effect on radiation-induced DNA double-strand breaks in the human fibroblast HF19 cell line. Int J Radiat Biol 69: 241–249. 8609461 10.1080/095530096146084

[96] Foray N, Charvet A-M, Duchemin D, Favaudon V, Lavalette D (2005) The repair rate of radiation-induced DNA damage: A stochastic interpretation based on the Gamma function. J Theoret Biol 236: 448–458. 15975603 10.1016/j.jtbi.2005.03.027

[97] Banath JP, Fushiki M, Olive PL (1998) Rejoining of DNA single- and double-strand breaks in human white blood cells exposed to ionising radiation. Int J Radiat Biol 73: 649–660. 9690683 10.1080/095530098141906

[98] Trseciak AR, Barnes J, Ejiogu N, Foster K, Brant LJ, et al (2008) Age, sex, and race influence single-strand break repair capacity in a human population. Free Radic Biol Med 45: 1631–1641. 18845243 10.1016/j.freeradbiomed.2008.08.031 PMC3072751

[99] Alaoui-Jamali MA, Batist G, Lehnert S (1992) Radiation-Induced Damage to DNA in Drug- and Radiation-Resistant Sublines of a Human Breast Cancer Cell Line. Radiation Res 129: 37–42. 1728055

[100] Cowell IG, Sunter NJ, Singh PB, Austin CA, Durkacs BW, et al (2007) γH2AX Foci Form Preferentially in Euchromatin after Ionising-Radiation. PLoS ONE 2: e1057. 17957241 10.1371/journal.pone.0001057 PMC2020439

[101] Falk M, Lukasova E, Gabrielova B, Ondrej V, Kosubek S (2008) Local changes of higher-order chromatin structure during double strand break repair. J. Phys: Conference Series 101: 012018.

[102] Lin A, Wang S, Nguyen T, Shire K, Frappier L (2008) The EBNA1 protein of Epstein-Barr virus functionally interacts with Brd4. J Virol 82: 12009–12019. 18922874 10.1128/JVI.01680-08 PMC2593323

[103] Shin C-G, Strayer JM, Wani MA, Snapka RM (1990) Rapid Evaluation of Topoisomerase Inhibitors: Caffeine Inhibition of Topoisomerases In Vivo. Teratog Carcinog Mutagen 10: 41–52. 1971968 10.1002/tcm.1770100106

[104] Machida K, Cheng KT, Sung VM, Shimodaira S, Lindsay KL, et al (2004) Hepatitis C virus induces a mutator phenotype: Enhanced mutations of immunoglobulin and protooncogenes. Proc Natl Acad Sci USA 101: 4262–4267. 14999097 10.1073/pnas.0303971101 PMC384729

[105] Trivedi P, Cuomo L, de Campos-Lima PO, Imreh MP, Kvarnung K, et al (1993) Integration of a short Epstein-Barr virus DNA fragment in a B95–8 virus converted Burkitt lymphoma line expressing Epstein-Barr nuclear antigens EBNA2 and EBNA5. J Gen Virol 74: 1393–1398. 8393080 10.1099/0022-1317-74-7-1393

